# Reply to Dr. C. N. Pierce

**Published:** 1897-07

**Authors:** 

**Affiliations:** The Odontological Society of Pennsylvania


### REPLY TO DR. C. N. PEIRCE.

To the Editor:

Sir,—In the June number of the International Dental Journal appeared an article signed by Dr. C. N. Peirce. The resolution he publishes as having been passed by the Society was offered by him, but was defeated in the discussion. The official facts of the case are these:

The Odontological Society of Pennsylvania held a special meeting November 10, 1896, for the purpose of hearing the report of a committee previously appointed to investigate the election irregularity said to have been perpetrated at the Pennsylvania State Dental Society, at its annual meeting, held at Bellefonte last July.

The report as adopted by the Odontological Society would seem to clearly prove that an irregularity had been committed. According to the law of precedent of the Odontological Society, on request, I furnished reprints of the proceedings of that special meeting, which were unofficially circulated for the information of any member of the profession who might be interested.

As the Editor of the Odontological Society, I wish to state clearly that I prepared the document in question, that it is a true reprint from our proceedings.

The motion which was passed as a substitute for the one offered by Dr. Peirce, and which I seconded, merely requested the editor to publish a disclaimer for the Society of official responsibility for the distribution of the said pamphlets.

Joseph Head,

*Editor of the Odontological Society of Pennsylvania.*

